# The role of the general practitioner in multidisciplinary teams: a qualitative study in elderly care

**DOI:** 10.1186/s12875-018-0726-5

**Published:** 2018-03-10

**Authors:** Sietske M. Grol, Gerard R. M. Molleman, Anne Kuijpers, Rob van der Sande, Gerdine A. J. Fransen, Willem J. J. Assendelft, Henk J. Schers

**Affiliations:** 1 Department of Primary and Community Care, Radboud University Medical Center, route 119, PO Box 9101, 6500 HB Nijmegen, the Netherlands; 2 Department of Healthy Living, Community Health Service Gelderland-Zuid, POB 1120, 6501 BC Nijmegen, the Netherlands; 3 Lectureship of primary and community care, Han University of Applied Sciences, POB 6960, 6503 GL Nijmegen, the Netherlands

**Keywords:** Focus groups, Frail older people, Leadership, Integrated health care systems, Qualitative research, General practitioner, Multidisciplinary team meetings

## Abstract

**Background:**

In the western world, a growing number of the older people live at home. In the Netherlands, GPs are expected to play a pivotal role in the organization of integrated care for this patient group. However, little is known about how GPs can play this role best. Our aim for this study was to unravel how GPs can play a successful role in elderly care, in particular in multidisciplinary teams, and to define key concepts for success.

**Methods:**

A mixed qualitative research model in four multidisciplinary teams for elderly care in the Netherlands was used. With these four teams, consisting of 46 health care and social service professionals, we carried out two rounds of focus-group interviews. Moreover, we performed semi-structured interviews with four GPs. We analysed data using a hybrid inductive/deductive thematic analysis.

**Results:**

According to the health care and social service professionals in our study, the role of GPs in multidisciplinary teams for elderly care was characterized by the ability to ‘see the bigger picture’. We identified five key activities that constitute a successful GP role: networking, facilitating, team building, integrating care elements, and showing leadership. Practice setting and phase of multidisciplinary team development influenced the way in which GPs fulfilled their roles. According to team members, GPs were the central professionals in care services for older people. The opinions of GPs about their own roles were diverse.

**Conclusions:**

GPs took an important role in successful care settings for older people. Five key concepts seemed to be important for best practices in care for frail older people: networking (community), facilitating (organization), team building (professional), integrating care elements (patient), and leadership (personal). Team members from primary care and social services indicated that GPs had an indispensable role in such teams. It would be advantageous for GPs to be aware of this attributed role. Attention to leadership competencies and to the diversity of roles in multidisciplinary teams in GP training programmes seems useful. The challenge is to convince GPs to take a lead, also when they are not inclined to take this role in organizing multidisciplinary teams for older people.

## Background

There is growing emphasis on teamwork and the role of GPs in multidisciplinary teams [1–3]. This applies especially to care for older people in the community [4]. The population of older people living at home is growing substantially across leading western countries [5, 6]. People live longer, resulting in multimorbidity and frailty, often combined with social problems [7]. Frailty is defined as the accumulation of deficits and diminishing reserves [8]. Thus, professionals in care and social services are faced with growing demands. This provides an impetus for those professionals to collaborate in new configurations, in order to enable older people to live at home as long and healthy as possible [9]. According to the OECD, the care and social services should align their actions, starting with defining the needs of frail older people [10]. Based on these needs, professionals should aim to deliver patient-centred integrated services [11–13]. Integrated models of care seem to hold the potential to meet economic, health, and social care challenges of ageing populations [14]. Integrated care is defined as a means to improve access, quality, and continuity of services in a more efficient way, especially for people with complex needs [15–17].

GPs provide care relating to a wide variety of patient problems, based on relationships developed over time. Moreover, GPs are potentially in the position to play a role of importance in integrated, multidisciplinary teams in the community, such as in care for older people. They initiate diagnosis and treatment and may play a central role in care planning. However, not all GPs are able to play this role [18–20]. Multidisciplinary team configurations of health and social care professionals differ and GPs roles vary widely depending on circumstances [21–24]. It is largely unknown which factors determine the uptake of a successful role by GPs in integrated models of care. In literature, little is found about how GPs shape their role in care for older people, and evidence is lacking about the best way to fulfil this role [25–27]. Most literature about the role of the GP is disease specific [25, 28, 29]. Therefore, the goal of the study was to obtain key lessons about the uptake of GP roles in successful multidisciplinary teams for elderly care. Our research question therefore was: What is the role of GPs in successful integrated multidisciplinary teams for elderly care?

### Context of the study

This study was part of a project called PRECURO, a larger study focusing on the increase of insight into the way integrated care (medical care, social services, and community health services) is organized for vulnerable older people living at home, and which hindering and stimulating factors play a role. In addition, specific attention was paid to the experience of older people receiving care and social services and the ways in which these experiences could be used to improve multidisciplinary care. The study was carried out in 2013 and 2014 in four multidisciplinary teams in the region of Nijmegen, the Netherlands. In this study, we used a participatory action research (PAR) approach. This type of qualitative research is characterized by a cycle of reflection, data collection, and action that aims to improve health and care through involving health care providers. They, in turn, take action to improve the care and services that they provide [30]. Data collection took place between February 2013 and June 2015 and consisted of interviews, focus-group meetings, document analysis, observations, and exchange meetings (for a timeline of the project PRECURO, see Appendix 1).

## Methods

For this study, we organized two rounds of four focus-group meetings and four individual interviews with GPs in four multidisciplinary teams [31–36]. We followed the consolidated criteria for reporting qualitative research (COREQ) [37–40] and used various methods of triangulation: methodological, data, theoretical, and investigator triangulation [41]. We applied member checking during two feedback meetings with the multidisciplinary teams, during which we presented the outcomes of the interviews and focus-group meetings.

### Participants of focus groups

In the Netherlands, all patients are registered with a GP, who on average deals with more than 95% of presented medical problems and arranges referral to secondary care when needed. A generalist and patient-oriented approach and continuity of care are features of Dutch GP services. The GP coordinates care for frail older patients with complex needs [42, 43]. We recruited four GP best practices in care for older people. We chose this focus because we expected to find successful GP roles in these multidisciplinary teams. The predicate ‘best practice’ was awarded on the basis of informal grounds. HS (a GP and senior researcher) and GM (a psychologist and community health scientist), both employed by the Department of General Practice of the Radboud University Medical Center and members of the research team, selected the GPs. Their selection was based on a general impression of elderly care performance by the GP. In the opinion of HS and GM, all GPs performed well. During the selection process, they used the following informal selection criteria: population served (deprived, commuter, city, village), years of experience with integrated elderly care, and size of the GP practice setting. The number of participating GPs was limited due to restricted resources. Heterogeneity was sought in terms of geography, preferred health care insurer, community characteristics, scale of general practice setting, and experience of GPs with integrated elderly care (*purposive sampling*) [44, 45]. All participating GPs, including their most important stakeholders (Table 1), aimed to organize integrated care for frail older people but differed in their phase of development. One general practice had been organizing integrated care for over 50 years, two other general practices since 2 years, and one general practice was just starting a multidisciplinary team meeting (MTM) procedure, based on a care programme for frail older people.

**Table 1:** Features of multidisciplinary teams and multidisciplinary team meetings

	Team 1 (*N* = 11)	Team 2 (*N* = 9)	Team 3 (*N* = 13)	Team 4 (*N* = 11)
Caregivers, attending focus-group interviews	1 GP	1 GP	1 GP	2 GPs
	1 practice nurse	3 community nurses	2 practice nurses	2 practices nurses
	3 community nurses	2 NHPSs	3 community nurses	2 community nurses
	2 physiotherapists	1 occupational therapist	1 mental health nurse	2 NHPSs
	1 occupational therapist	1 welfare worker	2 physiotherapists	2 welfare workers
	2 welfare workers	1 health broker	1 welfare worker	1 centre manager
	1 dietician		1 health broker	
Service area multidisciplinary team	4800 patients	2150 patients	7000 patients	38,000 patients
Demographic features	Village with 22,555 inhabitants, 15% = 65+ years	Deprived neighbourhood in a medium-sized city with 168,292 inhabitants, 14% = 65+ years	Commuters; neighbourhood with old village centre in medium-sized city with 168,292 inhabitants, 14% = 65+ years	Small town with 41,775 inhabitants, 15% = 65+ years
GP setting	General practice	General practice	Health centre	Health centre
Features of the MTM				
*Date of foundation*	2014	2011	1961	2011
*MTM chairperson*	GP	GP	GP	NHPS
*Target group*	Frail older people, 65+ years	Frail older people, 75+ years, exceptions allowed	Frail older people, 70+ years, exceptions allowed	Frail older people, 65+ years
*Submission of cases*	Screening	Screening	Case-finding	Case-finding
*Follow-up*	Case manager, care plan	Case manager, care plan, minutes, EPR, Care and Welfare Information Portal (ZWIP)	Case manager, minutes, EPR, Care and Welfare Information Portal (ZWIP)	Care plan, EPR
*Funding*	Research subsidy	Care programme by health insurer	Health insurer, funding for health centres	Research subsidy, local community health services
*Coordination*	Practice nurse	Community nurse	Practice nurse	Practice nurse
*Frequency*	1 × 3 months	1 × month	1 × month	On indication

*EPR* electronic patient record, *GP* general practitioner, *MTM* multidisciplinary team meeting, *NHPS* nursing home physician specialist, *ZWIP* Zorg- en WelzijnsInfoPortaal

To start, the principal investigator (SG, a health scientist and policy consultant) and GM interviewed the participating GPs individually, during which a stakeholders analysis was carried out. The GPs were asked about the most important collaboration partners in the organization of integrated elderly care. These so called stakeholders, often participants of MTMs, were approached for participation in our focus-group study (minimum six, maximum eight). GPs and their stakeholders together formed the so called ‘multidisciplinary teams’ for our study (Table 1).

### Focus groups, interviews and topic list

Each multidisciplinary team participated twice in a focus-group interview: once in 2013 and again in 2014. All participants consented via a written statement. We ensured anonymity and confidentiality by removing names from transcripts and replacing them by the name of the profession. The focus groups were moderated by SG. The focus-group meetings lasted 90–120 min and were minuted, audio taped, and transcribed verbatim. SG and AK (a biomedical scientist and junior researcher) read and corrected all transcripts. Results from the focus groups were discussed with the research team and presented and discussed with the multidisciplinary teams during exchange meetings after the focus-group interviews, as part of the reflective phase of PAR. The outcomes were documented and used as input for the next action-cycle research and for data analysis.

The semi-structured topic list for focus groups and interviews was based on a theoretical framework prepared on the basis of the ‘Healthy ALLiances (HALL) framework’ and on ‘Preconditions for healthy care: integrated and effective support by caregivers at lifestyle changes’ as prescribed by the Dutch Inspectorate for Healthcare (Appendix 2) [46, 47]. The theoretical framework addresses organizational, personal, and institutional factors of collaboration in primary care and public health and is available from the author.

The main question in the first focus-group interviews was: ‘How do the members of the multidisciplinary team work together, with the aim to improve care for older people in the community, in particular the connection between prevention, care, and welfare?’. Some specific questions were asked about the role of the GP such as ‘can you describe the role of the GP in the development of integrated care for older people?’ and ‘can you describe the influence of collaboration between the GP and the practice nurse on the development of integrated care of older people?’. We also asked the teams to start a quality-improvement project for better-organized integrated elderly care in their community. The teams were free to fill in the projects in their own way. In the second round of focus groups, we specifically addressed questions about the role of the GP in organizing integrated care and MTMs. We asked the teams questions about the quality-improvement projects that they had started. More questions than the question addressed in this paper were examined (see Appendix 2).

All focus-group meetings were observed by two to four research team members and discussed afterwards. The topic list was adjusted for each focus group due to differences in context and development of the multidisciplinary team. For instance, in the first round of focus groups, each team was asked to start a quality-improvement project. In the second focus group, specific questions about that project were asked.

The interviews were conducted by SG and GM. The interviews lasted about 60 min and were minuted by SG. The main questions for the interviews was ‘How is care for frail older people organized at this moment? What are your expectations of this research project? Who are your most important collaboration partners in the organization of integrated care for older people?’

### Coding and analysis

The theoretical framework was applied to a thematic analysis of all results. Analysis of the focus groups started at the end of the first round of focus groups and was supported by Atlas.ti version 7.1 software. The analysis involved both pragmatic initial coding and thematic coding based on the factors from the theoretical framework [48–51]. Two researchers (SG and AK) coded all data blinded to increase reliability. Parts of the transcripts were blindly coded by a third researcher, GF (a dietician and health scientist). Differences in coding and thematic interpretation were discussed and resolved, further reducing potential for researcher bias, until consensus was reached. To perform axial coding, transcripts were read and re-read and codes were altered and added. In the next phase, codes were collated into themes in the light of emerging analytical insights [52]. SG, GM, and HS discussed and reduced the themes to major themes towards the latter stages of data collection.

## Results

### Five major themes

Representatives of four multidisciplinary teams participated in the focus groups (Table 1). Two teams had been constructed around general practices, and two had been formed around health centres that contained general practices. The teams varied in number and profession of the participants, size and nature of the population served, experience with MTMs, and level of organization. One team had just started implementing an MTM, whereas the three other teams were more experienced with MTMs. All teams consisted of at least one GP, one practice nurse or community nurse, and one social worker. Other professionals that participated in one or more teams were nursing home physician specialists (NHPSs), physiotherapists, occupational therapists, health brokers, mental health nurses, and health-centre managers.

The outcomes of the first round of focus-group interviews could be characterized as information about the working methods of the MTMs in general and of the GP in particular. The second round of focus-group interviews provided more in-depth information about the role of the GP in integrated care for older people. In addition, we have collected information about quality-improvement projects. One team had used the time between the first and second focus-group interview to start the MTM and to map out all older adults in the community. The other three teams had worked on improving their working methods on the basis of the outcomes of the first focus-group interviews and the interviews with patients from their community.

Five key concepts about the roles that GPs play in these successful care teams were identified. They all fitted in the feeling of the participants of the MTMs that ‘*the GP sees the bigger picture’.* Attitudes and beliefs of GPs and their team members were interpreted within these key concepts (Table 2):

1. Networking
2. Facilitating
3. Team building
4. Integrating care
5. Leadership.

**Table 2:** GPs’ key concepts for multidisciplinary elderly care teams – *‘GPs see the bigger picture’*

	1. Key concepts		Starting teams		Experienced teams
1.	Networking *(community level)*	→	Establishing contacts with community partners, health insurers, hospitals, social services	→	Developing and maintaining contacts
2.	Facilitating *(organizational level)*	→	Choosing an EPR, negotiating with health insurers and social and care services, setting up an MTM	→	Adjusting MTM to demands of time, quality improvement
3.	Team building *(professional level)*	→	Team composition (type of professionals, selection of organizations, competencies, personality), distribution of tasks and responsibilities, improve connection among professionals	→	Encouragement of team members, equivalence between team members and GP(s)
4.	Integrating care *(patient level)*	→	Coordination of care in the medical domain, keeping an overview of care, connecting domains (hospitals, primary care, nursing homes, social services, community services, prevention)	→	Prevention of decline / preservation of functioning, keeping an overview, delivering proactive care.
5.	Leadership *(personal level)*	→	Passion for care for older people, clear vision, endurance, drive, taking responsibility	→	Focusing on medical domain, background position, relying on skills of team members, coordination of care

*EPR* electronic patient record, *GP* general practitioner, *MTM* multidisciplinary team meeting

Opinions of team members about the role of GPs did not differ much across teams. According to team members, GPs are the pivotal professionals in integrated elderly care, because they coordinate the medical domain and have the ability ‘*to see the bigger picture’*. Opinions of GPs about their own role differed. While two GPs considered themselves the central person in the care of the vulnerable older people, two other GPs found themselves of secondary importance.

### Networking

In all teams, partners in the community saw GPs as the spokesperson for professionals in primary care for older people. In the community, GPs encountered representatives from hospitals, municipalities, health insurers, and managers from care and social services. Either these organizations invited GPs to discuss collaboration in care for older people or GPs themselves took the initiative:

GP: It is nice that soon [...] we will have a meeting with the municipality where we will discuss a lot of problematic cases, not just the frail older people but also youngsters and multiproblem families, you name it. They are being discussed in order to decide who does what. And who is responsible for what, and how this will be organized. [Focus group 4.]

GPs, and sometimes NHPSs, took the lead in connecting primary and secondary care, social services, and the community. None of the other professionals in the four teams took a role in networking at this strategic level. Only physicians seem to take on this role. All team members networked at an operational level, in the meaning of working together in practice. Especially nurses were characterized as networkers or ‘spiders in the web’.

How GPs fulfilled the networking role differed among GPs. In the two general practices, GPs took the role of strategic networker. In the two health centres, GPs shared this role with managers and other GPs.

### Facilitating

The most important facilitating factors for integrated elderly care were the use of an electronic patient record (EPR), organization of MTMs, finance, and setting. Concerning all factors, GPs were determinative. GPs and team members of all four teams had the same opinions on this matter.

GPs decided what EPR was selected and who got access to it. In all teams, GPs, together with a nurse, organized the MTMs. Other team members characterized them as the logical pair to organize the MTM:

Social worker: The division of roles is fine this way. That has also to do with this lady [community nurse] doing the screening. She has the first information. That is very important. And she has contact with the GP about how to go on from there. It all starts with this sort of contact. In the multidisciplinary team meeting, we discuss about what aspects can be taken over. [Focus group 6.]

Finances for multidisciplinary consultation were insecure. Talk time is almost never funded. For GPs, it was sometimes possible to obtain extra funding from a health insurer or a research fund. For other team members, participation was mostly without extra funding:

Practice nurse: I think [...] we should be well-rewarded for the multidisciplinary team meeting. If you look at what kind of work you deliver and what comes out.

Physiotherapist: It is not paid.

Social worker: Welfare is an exception. This is social work, we do not have to justify it.

Mental health nurse: Can you claim the costs for the patients you discuss?

GP: No, we cannot. Of course, ultimately the GPs pay for it anyway.

Practice nurse: This is done basically to improve collaboration.

GP: I see also the benefits for the patient, so I think, looking at the bigger picture, it also saves work. [Focus group 2.]

The setting of the team played an important role facilitating the MTM. Both primary care centres had managers who supported the integrated elderly care development (e.g. organizing project teams, negotiating with health insurers). Besides this, three out of four teams had other social and health care professionals working in the same building, which was experienced as helping:

GP: Knowing who you are, who you need to speak to. We surely have the advantage of having all disciplines working under one roof and knowing the physiotherapists. That's really a big plus. We as GPs are very well connected with all disciplines. And now it is still better again, with the community and social services in the same building. [Focus group 5.]

### Team building

GPs took the lead in building multidisciplinary teams. MTMs were the core of integrated elderly care. All GPs selected the team members. In the more experienced teams, the participants discussed the composition of the MTM together. In the starting team, the GP decided solely the composition of the team:

GP: We are ultimately the director of the multidisciplinary team meeting. And I think I must stay ultimately responsible. But I also have to delegate more.

Physiotherapist 1: You [the GP] must remain in the pivotal position there.

GP: That is also prescribed by the health care insurance. This is really a requirement. And the community nurse. They facilitate in making care plans and providing case management. [Focus group 5.]

Team building also means to enhance the effectiveness and mutual binding of a team. Two factors stood out while observing the process of team building: distribution of roles and tasks and the degree of equivalence of team members:

GP: We do it as a group. You do not have to lean on one profession, you divide tasks.

Community nurse: That is what you want to accomplish, removing the barriers. We look at who is most suitable for the question at hand.

GP: I think we have always had the same kind of people. We are all people who take action. When we are frustrated, someone else takes over. And also, perhaps, a key element is that we have collected people around us with whom we collaborate easily, with whom we are connected well, and who are trustworthy. [Focus group 3.]

In the starting team, the GP and practice nurse performed all tasks. The other teams showed more division of tasks among team members. GPs took a step back and focused more on the medical domain. At the same time, they kept the bigger picture in sight. In those teams, more equality among team members was observed:

NHPS: Because it is important that you are working with a well-organized group within a certain area. You know where to find each other, you know each other's strengths and capabilities.

GP: That, and that there is equivalence, among team members. That is also important. [Focus group 3.]

Team building at the start was seen as a task solely for the GP. As the situation developed, the GP still kept the overview and took initiatives, but team building became more a responsibility of the team as a whole.

### Integrating care

In all teams, GPs were, together with nurses, the ones to integrate most actions of the multidisciplinary team. GPs as well as team members experienced that working as a team improved the connection between each other. In more experienced teams, team members felt able to deliver better quality of care to patients:

GP: You start really medically, and after two multidisciplinary team meetings, the medical stuff is in order and then we talk about care so much more. So, during the first multidisciplinary team meetings, I do the talking mostly and then I am actually unnecessary. Then we talk about care and not about the pills. That's all in order. Those underlying somatic problems are in order. And then patients have mainly requests for help, because it is still ultimately about the care needs of those clients or carers, in the area of care and welfare. [Focus group 3.]

As teams existed longer, they were better able to deliver proactive care and prevent crises and decline of functioning in daily life:

GP: I have the feeling that I have more to offer for the frail older people with complex needs. There are now all kinds of options to cover my blind spots. I have more, so I can offer people more. Not only for patients, but especially for caregivers. I am less often powerless, empty handed. The broad, holistic medicine, with the other disciplines in the multidisciplinary team meeting, is better addressed now. And secretly I think I deliver better care. I now have a better view of my frail older patients and I often know in advance when someone is in danger of ending up in a crisis. If it happens, it is wonderful that they are already charted and there is a care indication. [Focus group 6.]

GPs coordinated the medical domain, ensuring that all physical aspects were addressed. They coordinated, in two teams together with the NHPSs, the follow-up action by the community or practice nurse in order to investigate all life domains of the patient (e.g. social, mental, functioning in daily life) during a home visit. During the MTMs, we observed the GPs connecting these domains. This happened more often in experienced teams:

GP: Yes, one person puts the patient on the agenda and asks questions. Then we discuss as a team if there are any ideas or additions, and we decide what needs to be done. The patient usually returns on the agenda next month or a few months later, and will be discussed again. Sometimes, another team member is being appointed. For example, ‘[Social worker] will you go and have a look and see what you think?’ [Social worker] will tell us what she saw. And then it is decided whether we should take further action or whether we can let the case rest. That is usually the structure. [Focus group 7.]

### Leadership

We observed GPs showing leadership in all teams. They were all passionate about delivering integrated, good-quality, person-centred care. Based on this drive, GPs initiated integrated elderly care:

NHPS: Well, look, it was also true that you [GP], for example, also had prolonged contact with the occupational therapist and you were well able to find social services before we even started this project. It's not just from this first multidisciplinary team meeting that everything is put in motion, […] and it is particularly the GPs and those who do the screening [community nurses] who are already so alert to what emerges. They say ‘no, we should do this now’ or ‘start home care’. That is what I see happening.

Social worker: But also how motivated you [GP] are. You are very passionate! [Focus group 3.]

Opinions from GPs about their own role differed. While some GPs saw themselves as a pivotal professional, others downplayed their role and emphasized the importance of others, especially nurses and social workers who are, in the GPs’ opinion, closer to patients:

GP: I'm still thinking about the notion that you cannot bypass the doctor in the integrated care process. I keep an eye on the medical stuff. I think you [home care, welfare] are more often at the people’s homes, seeing many more things. I think that I, as a GP, sit here as a coordinator and try to continue to see the bigger picture, but I'm certainly not indispensable. [Focus group 7.]

In more experienced teams, GPs were able to take a position in the background, relying on the skills and added value of team members.

## Discussion

### Summary of results

In this study, we found that GPs who followed best practices showed leadership to initiate and improve the quality of integrated elderly care by building successful multidisciplinary teams. GPs were considered, by their team members, to be the professionals in primary care with the best overview, and they were perceived to connect care and welfare organizations, facilities, professionals, and health domains and to combine personal vision with initiative and endurance. The key elements of successful GPs’ roles in integrated elderly care teams were defined: networking (community), facilitating (organization), team building (professional), integrating care (patient), and leadership (personal). GPs practised their role differently, influenced by practice setting and the developmental stage of integrated elderly care. GPs had been more dominant in teams that had just started. In experienced teams, GPs shared roles and responsibilities with other team members, because they were more familiar with their knowledge, skills, and added value, resulting in more equivalence among team members.

### Comparison with existing literature

Comparing our results with the three clusters of the Healthy ALLiances framework [46], used in our theoretical framework, we found that, looking at the *institutional cluster*, GPs and NHPSs were active in building networks at a strategic level, developing integrated care programmes with primary care groups, and applying for funding. In the *organizational cluster*, GPs facilitated integrated care, in close collaboration with nurses. Sharing patient information through the use of an EPR played an important role as well as ‘building on capacities’, meaning relying on each other’s skills and expertise. Considering the *personal cluster*, three factors were helpful to establish integrated elderly care: positive attitudes and beliefs towards collaboration, personal relationships among team members, and self-efficacy of GPs about their own capability to make a difference in the team. The amount of self-efficacy differed among GPs and influenced their way of performing on the five key concepts of this study.

In position papers, physicians are urged to reform health care to more sustainable systems [53, 54]. Some advocate that GPs should take the lead in reforming primary care [9, 55, 56]. Our study shows that GPs in successful teams did indeed take the lead in reforming primary care for older people. Stakeholders in the community saw GPs as natural leaders. Herzog et al. stated that a conceptual framework that provides guidance to GPs concerning their specific role in care for older people is needed [26]. Our study provides the building bricks for such a guide and addresses five key concepts in which GPs have a unique role to play.

Literature on the role of GPs in multidisciplinary primary care teams, such as care for older people, are scarce [57]. Only a few reports address the role of GPs in care for the frail older people. For instance, George et al. claimed that current reforms in primary care for older people require GPs to remain generalists and to be less of a gatekeeper and more of a care navigator, working alongside specialists and with allied professionals in care and welfare, to provide more care in the community [58]. The Kings Fund questioned whether GPs are able to deliver the support that older people with complex needs require [4]. In its opinion, GPs often seem to play a tangential rather than central role in care coordination for older people. In our study, GPs initiated the process of care coordination, whereas other team members, mostly nurses or social workers, carried out this task. Notwithstanding their limitations, our findings do suggest that experienced and motivated GPs are indeed capable of organizing integrated care teams for older people, together with partners in primary care.

The findings in this study raise questions about the extent to which the GPs’ role in initiating integrated elderly care can be considered a core competence that can be trained. Not all GPs have a passion for organizing multidisciplinary teams or care for older people. However, many training programmes for physicians and GPs around the world are based on the CanMEDS Competency Framework, with seven roles for physicians. The terminology of the ‘Manager’ role was recently changed to ‘Leader’ [59, 60]. The main motivation for this change was the increasing complexity of health care and the fact that physicians are often ill trained to perform leadership in current practice context [61]. This call for leadership corresponds with our findings. There is strong evidence that, in many GP training programmes as well as medical curricula, the emphasis lies on the ‘Medical Expert’ and ‘Communicator’ roles [62, 63]. In order to enable GPs to play a leading role in reforming primary care, more emphasis should be put on the Leader role as well.

### Strengths and limitations

We used mixed qualitative research to obtain the answers to our study question. Data were collected using different research instruments: in-depth interviews with GPs and two rounds of focus groups. This methodological triangulation resulted in both variation and data saturation. Analysis was carried out based on a systematically developed theoretical framework, without losing an open mind towards the interpretation of the results.

Limitations of this study are the nature and size of our sample and the local setting. However, we purposefully studied best practices in order to be able to learn more about their success factors. It was deliberately not the intention to carry out a representative study. We used an informal selection of well-performing practices, which made the outcomes diverse and showed a broad spectrum of care models for frail older people in the Netherlands. However, we have handed out the predicate ‘best practice’ by ourselves in a non-objective way.

GPs were asked to choose their most important stakeholders. This choice of perspective, as well as the setting of the study in Dutch health care, may hinder broad applicability of our results. Further research is needed to determine whether our key concepts for successful operation of GPs in integrated care are generalizable to physicians in other settings or health care systems.

The participatory action research approach, used in the larger study of which this study was part, may have influenced the outcomes between the first and second focus-group interviews. Between the first and second focus-group interview, the teams worked on the improvement of their elderly care supply and participated in two exchange meetings with the four multidisciplinary teams. The results of the second focus-group meeting were therefore definitely influenced by both the outcomes of the first focus group, the quality improvement project and the inspiration that the teams had gained in the exchange meetings. The fact that this happened is a typical characteristic of PAR: improvement of care through participation in research.

### Implications for education, practice and research

GPs’ key concepts for multidisciplinary elderly care teams will probably be relevant for countries where the GP has a similar pivotal position as in the Netherlands, such as in Scandinavian countries, the United Kingdom, and Canada. Our findings will gain strength by studying successful multidisciplinary teams in other western countries, preferably with a PAR approach. This will probably lead to deepening and widening of the five key concepts, including the possibility to deliver practical guidance for GPs in different phases of the development of integrated care for frail older people in the community. Further research is needed on the uptake of our key concepts in GP training programmes. Attention to leadership, networking, facilitating, team building, and integrating care elements in GP training programmes seems useful.

## Conclusions

In the opinions of health care and social professionals in our study, GPs should not be hesitant to take the initiative to organize multidisciplinary teams for the care of older people with complex needs. In our opinion, GPs should be more aware of this professional pivotal position in the community, as regarded by other professionals in primary care and social services. A curriculum on organizing multidisciplinary teams, and specifically on leadership, in training (or re-training) programmes could provide tools for GPs to carry out this role. Our key concepts could provide guidance herein. Differences among GPs, as observed in this study, should be taken into account. While some GPs will take the lead naturally, others will be more hesitant, due to different causes: underestimation of their importance as a medical doctor in integrated elderly care, insecurity about their own competencies as a leader and collaborator, or lack of motivation. For the first group, a curriculum on building multidisciplinary teams could be helpful. For the second group, this will probably not be enough. Of course, we can look for ways to support GPs to play their role in collaboration. However, the question that precedes this is whether GPs have a choice to take the lead in multidisciplinary team building or not. Is it not the case that organizing multidisciplinary care for groups of patients with complex health and care problems is part of the GP profession? This is a question that should be answered by the occupational group.

## Figures and Tables

**Fig. 1:**
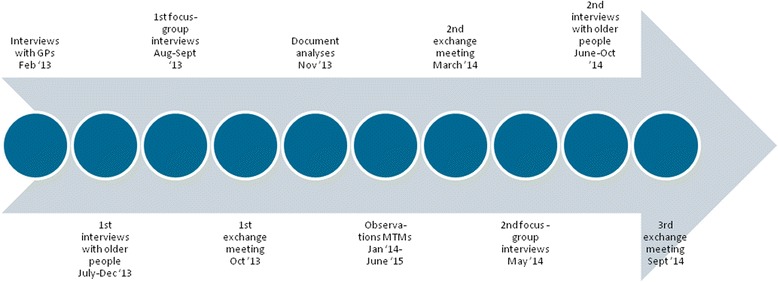
Time line of the PRECURO participatory action research

## Appendix 2

**Table 3:** Topic list of focus groups and interviews

Category	Questions
Institutional factors1.1.1.1.1.1.1.1.1.1.	1. Is there joint policy concerning integrated elderly care? Are objectives defined concerning integrated elderly care?
	2. In what way are patients involved in the organization of care?
	3. Is the target group of older people delineated? Was screening or case finding used in the elderly population?
	4. Who are the stakeholders of the GPs in integrated care for the elderly population?
	5. In the multidisciplinary team, what agreements are in writing concerning the organization of integrated care for older people?
	6. What are the effects of structural consultation among professionals from care and welfare organizations?
	7. What are the effects of health care professionals working under one roof?
	8. Does consultation between the multidisciplinary team and care insurers regarding older people take place? What agreements have been made on the funding of integrated elderly care? Is finance of influence on the partnership? In what way?
	9. Does consultation between the multidisciplinary team and the municipality regarding older people take place? In what way?
	10. Is there systematic evaluation and improvement?
	11. Is available knowledge being used? (Guidelines, protocols.)
(Inter)personal factors1.1.1.1.1.1.	12. What are the differences in values, beliefs, and attitudes concerning (a) the integrated elderly care team and (b) the other network partners?
	13. Self-efficacy: do professionals feel confident about themselves and each other as collaborator?
	14. Social identity: do partners also perceive a shared identity?
	15. Nature of interpersonal relationships: do partners get along well with each other as collaborators?
	16. Can you describe the role of the GP in the development of integrated elderly care?
	17. Can you describe the role of the NHPS in the development of integrated elderly care?
	18. Are there any agreements on taking the lead/control in integrated elderly care?
Factors concerning the organization of multidisciplinary teams	19. Flexible timeframe: is time and flexibility available for the development of integrated elderly care? How do you make time?
	20. How is (multidisciplinary) consultation organized in the multidisciplinary team? (Frequency, composition, agenda, etc.) Are MTMs minuted? Are agreements transferable to colleagues?
	21. Shared mission: did partners agreed on a shared mission, goals, and plan of action?
	22. Do team members agree on distribution of roles and responsibilities?
	23. Can you describe the influence of the collaboration between the GP and the practice nurse on the development of integrated elderly care?
	24. Do team members build on each other’s capacities?
	25. Are facilities for formal and informal communication in place?
	26. What are the characteristics of the management of the multidisciplinary team (neutral, facilitating, empowering)?
	27. Are the results of the personal and the team effort to the team members visible?

*GP* general practitioner, *MTM* multidisciplinary team meeting, *NHPS* nursing home physician specialist

## Abbreviations

CANMeds: Canadian Medical Education Directives for Specialists
EPR: Electronic patient record
GP: General practitioner
MTM: Multidisciplinary team meeting
NHPP: Nursing home physician specialist
PAR: Participatory action research
ZWIP: Zorg- en WelzijnsInfoPortaal

## References

[1] Mulvale G, Embrett M, Razavi SD (2016). ‘Gearing Up’ to improve interprofessional collaboration in primary care: a systematic review and conceptual framework. BMC Fam Pract.

[2] Pullon S, Morgan S, Macdonald L, McKinlay E, Gray B (2016). Observation of interprofessional collaboration in primary care practice: a multiple case study. J Interprof Care.

[3] Boeckxstaens P, De Graaf P (2011). Primary care and care for older persons: position paper of the European forum for primary care. Qual Prim Care.

[4] Goodwin N, Dixon A, Anderson G, Wodchis W. Providing integrated care for older people with complex needs. In: The kings fund; 2014. 10.5334/ijic.2249 PMC4628509 26528096

[5] OECD (2015). Ageing: debate the issues. OECD insights.

[6] Rechel B, Grundy E, Robine JM, Cylus J, Mackenbach JP, Knai C, McKee M (2013). Ageing in the European Union. Lancet.

[7] OECD (2011). Health reform: meeting the challenge of ageing and multiple morbidities.

[8] Clegg A, Young J, Iliffe S, Rikkert MO, Rockwood K (2013). Frailty in elderly people. Lancet.

[9] Plochg T, van den Broeke JR, Kringos DS, Stronks K (2012). Integrating primary care and public health. Am J Public Health.

[10] OECD (2015). Integrating social services for vulnerable groups.

[11] Chan H-T, Cheng S-J, Su H-J (2008). Integrated care for the elderly in the community. Int J Gerontol.

[12] Clemens E, Wetle T, Feltes M, Crabtree B, Dubitzky D (1994). Contradictions in case management: client-centered theory and directive practice with frail elderly. J Aging Health.

[13] Gilleard C, Hyde M, Higgs P. The impact of age, place, aging in place, and attachment to place on the well-being of the over 50s in England. Res Aging. 2007;29(6):1–16.

[14] MacAdam M (2008). Frameworks of integrated care for the elderly: a systematic review.

[15] Leutz WN (1999). Five laws for integrating medical and social services: lessons from the United States and the United Kingdom. Milbank Q.

[16] Valentijn PP, Schepman SM, Opheij W, Bruijnzeels MA (2013). Understanding integrated care: a comprehensive conceptual framework based on the integrative functions of primary care. Int J Integr Care.

[17] Kodner DL, Kyriacou CK. Fully integrated care for frail elderly: two American models. Int J Integr Care. 2000;1:1–19. 10.5334/ijic.11 PMC1533997 16902699

[18] Stange KC, Jaen CR, Flocke SA, Miller WL, Crabtree BF, Zyzanski SJ (1998). The value of a family physician. J Fam Pract.

[19] Katon W, Von Korff M, Lin E, Simon G (2001). Rethinking practitioner roles in chronic illness: the specialist, primary care physician, and the practice nurse. Gen Hosp Psychiatry.

[20] Schoenmakers B, Buntinx F, Delepeleire J (2009). What is the role of the general practitioner towards the family caregiver of a community-dwelling demented relative? A systematic literature review. Scand J Prim Health Care.

[21] Christensen K, Doblhammer G, Rau R, Vaupel JW (2009). Ageing populations: the challenges ahead. Lancet.

[22] Plochg T, Klazinga NS (2002). Community-based integrated care: myth or must?. Int J Qual Health Care.

[23] Trivedi D, Goodman C, Gage H, Baron N, Scheibl F, Iliffe S, Manthorpe J, Bunn F, Drennan V (2013). The effectiveness of inter-professional working for older people living in the community: a systematic review. Health Soc Care Community.

[24] Grant A, Mackenzie L, Clemson L (2015). How do general practitioners engage with allied health practitioners to prevent falls in older people? An exploratory qualitative study. Australas J Ageing.

[25] Lyngsø AM, Godtfredsen NS, Frølich A. Interorganisational integration: healthcare professionals’ perspectives on barriers and facilitators within the Danish healthcare system. Int J Integr Care. 2016;16(1):1–10. 10.5334/ijic.2449 PMC5015550 27616948

[26] Herzog A, Gaertner B, Scheidt-Nave C, Holzhausen M (2015). ‘We can do only what we have the means for’ general practitioners’ views of primary care for older people with complex health problems. BMC Fam Pract.

[27] Balasubramanian BA, Chase SM, Nutting PA, Cohen DJ, Strickland PA, Crosson JC, Miller WL, Crabtree BF, Team US (2010). Using learning teams for reflective adaptation (ULTRA): insights from a team-based change management strategy in primary care. Ann Fam Med.

[28] Hinrichs T, Brach M (2012). The general practitioner's role in promoting physical activity to older adults: a review based on program theory. Curr Aging Sci.

[29] Chicoulaa B, Balardy L, Stillmunkes A, Mourey L, Oustric S, Rouge Bugat ME (2016). French general practitioners’ sense of isolation in the management of elderly cancer patients. Fam Pract.

[30] Baum F, MacDougall C, Smith D (2006). Participatory action research. J Epidemiol Community Health.

[31] Wong LP (2008). Focus group discussion: a tool for health and medical research. Singap Med J.

[32] Sharts-Hopko NC (2001). Focus group methodology: when and why?. J Assoc Nurses AIDS Care.

[33] Lucasey B (2000). Qualitative research and focus group methodology. Orthop Nurs.

[34] Kitzinger J (1995). Qualitative research. Introducing focus groups. BMJ.

[35] Evers J (2007). Kwalitatief interviewen: kunst én kunde.

[36] Britten N (1995). Qualitative interviews in medical research. BMJ.

[37] Malterud K (2001). Qualitative research: standards, challenges, and guidelines. Lancet.

[38] Murphy E, Dingwall R, Greatbatch D, Parker S, Watson P (1998). Qualitative research methods in health technology assessment: a review of the literature. Health Technol Assess.

[39] Cohen DJ, Crabtree BF (2008). Evaluative criteria for qualitative research in health care: controversies and recommendations. Ann Fam Med.

[40] Tong A, Sainsbury P, Craig J (2007). Consolidated criteria for reporting qualitative research (COREQ): a 32-item checklist for interviews and focus groups. Int J Qual Health Care.

[41] Farmer T, Robinson K, Elliott SJ, Eyles J (2006). Developing and implementing a triangulation protocol for qualitative health research. Qual Health Res.

[42] LHV N (2012). Toekomstvisie Huisartsenzorg 2022. Modernisering naar een menselijke maat: Huisartsenzorg in 2022.

[43] Van Weel C, Schers H, Timmermans A (2012). Health care in the Netherlands. J Am Board Fam Med.

[44] Patton MQ (2002). Two decades of developments in qualitative inquiry a personal, experiential perspective. Qual Soc Work.

[45] Coyne IT (1997). Sampling in qualitative research. Purposeful and theoretical sampling; merging or clear boundaries?. J Adv Nurs.

[46] Koelen MA, Vaandrager L, Wagemakers A (2012). The healthy ALLiances (HALL) framework: prerequisites for success. Fam Pract.

[47] Inspectie Gezondheidszorg en Jeugd [Healthcare and Youth Inspectorate]. Randvoorwaarden gezonde zorg: geïntegreerde en effectieve ondersteuning door zorgverleners bij leefstijlverandering [Basic conditions for health care: integrated and effective care by health care providers in cases of lifestyle changes]. The Hague: Ministry of Care, Welfare and Sports; 2012.

[48] Corbin JM, Strauss A (1990). Grounded theory research: procedures, canons, and evaluative criteria. Qual Sociol.

[49] Glaser B, Strauss A (1967). The discovery of grounded theory: strategies for qualitative research.

[50] Evers J (2015). Kwalitatieve analyse: kunst en kunde.

[51] Saldaña J (2015). The coding manual for qualitative researchers.

[52] Boeije HR (2005). Analyseren in kwalitatief onderzoek: denken en doen.

[53] West M, Armit K, Loewenthal L, Eckert R, West T, Lee A (2015). Leadership and leadership development in healthcare: the evidence base.

[54] PMLUo T (2015). Raamwerk medisch leiderschap.

[55] Mostashari F, Sanghavi D, McClellan M (2014). Health reform and physician-led accountable care: the paradox of primary care physician leadership. JAMA.

[56] Goodwin N, Dixon A, Poole T, Raleigh V (2011). Improving the quality of care in general practice. Report of an independent inquiry commissioned by the King’s fund.

[57] Drewes YM, Koenen JM, de Ruijter W, van Dijk-van Dijk DJ, van der Weele GM, Middelkoop BJ, Reis R, Assendelft WJ, Gussekloo J (2012). GPs’ perspectives on preventive care for older people: a focus group study. Br J Gen Pract.

[58] George R, Hammett S, Siegel S, Hudson N, Jones DL, Colgan K (2014). Better care for frail older people. Working differently to improve care.

[59] Frank JR, Snell L, Sherbino J (2015). CanMEDS 2015 physician competency framework.

[60] Borleffs JC, Mourits MJ, Scheele F (2016). CanMEDS 2015: nog betere dokters?. Ned Tijdschr Geneeskd.

[61] Matlow A, Chan MK, Bohnen JD, Blumenthal DM, Sanchez-Mendiola M, de Camps MD, Samson LM, Busari J (2016). Collaborating internationally on physician leadership education: first steps. Leadersh Health Serv.

[62] Ng VK, Burke CA, Narula A (2013). Knowledge of CanMEDS-family medicine roles: survey of Canadian family medicine residents. Can Fam Physician.

[63] Whitehead CR, Austin Z, Hodges BD (2011). Flower power: the armoured expert in the CanMEDS competency framework?. Adv Health Sci Educ Theory Pract.

